# A Comparative Study on the Metabolism of *Epimedium koreanum Nakai*-Prenylated Flavonoids in Rats by an Intestinal Enzyme (Lactase Phlorizin Hydrolase) and Intestinal Flora

**DOI:** 10.3390/molecules19010177

**Published:** 2013-12-24

**Authors:** Jing Zhou, Yan Chen, Ying Wang, Xia Gao, Ding Qu, Congyan Liu

**Affiliations:** 1 Key Laboratory of New Drug Delivery System of Chinese Meteria Medica, Jiangsu Provincial Academy of Chinese Medicine, 100 Shizi Road, Nanjing 210028, Jiangsu, China; E-Mails: happyjingzhou@126.com (J.Z.); wangying9021@163.com (Y.W.); gaoxia0218@163.com (X.G.); quding1985@hotmail.com (D.Q.); liucongyan2007@126.com (C.L.); 2 College of Pharmacy, Jiangsu University, Zhenjiang 212013, Jiangsu, China; 3 College of Pharmacy, Nanjing University of Chinese Medicine, Nanjing 210046, Jiangsu, China

**Keywords:** lactase phlorizin hydrolase, intestinal flora, prenylated flavonoids, metabolite, *Epimedium*

## Abstract

The aim of this study was to compare the significance of the intestinal hydrolysis of prenylated flavonoids in *Herba Epimedii* by an intestinal enzyme and flora. Flavonoids were incubated at 37 °C with rat intestinal enzyme and intestinal flora. HPLC-UV was used to calculate the metabolic rates of the parent drug in the incubation and LC/MS/MS was used to determine the chemical structures of metabolites generated by different flavonoid glycosides. Rates of flavonoid metabolism by rat intestinal enzyme were quicker than those of intestinal flora. The sequence of intestinal flora metabolic rates was icariin > epimedin B > epimedin A > epimedin C > baohuoside I, whereas the order of intestinal enzyme metabolic rates was icariin > epimedin A > epimedin C > epimedin B > baohuoside I. Meanwhile, the LC/MS/MS graphs showed that icariin produced three products, epimedin A/B/C had four and baohuoside I yielded one product in incubations of both intestinal enzyme and flora, which were more than the results of HPLC-UV due to the fact LC/MS/MS has lower detectability and higher sensitivity. Moreover, the outcomes indicated that the rate of metabolization of flavonoids by intestinal enzyme were faster than those of intestinal flora, which was consistent with the HPLC-UV results. In conclusion, the metabolic pathways of the same components by intestinal flora and enzyme were the same. What’s more, an intestinal enzyme such as lactase phlorizin hydrolase exhibited a more significant metabolic role in prenylated flavonoids of *Herba Epimedi* compared with intestinal flora.

## 1. Introduction

**Figure 1:**
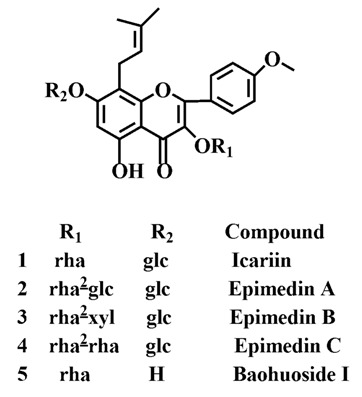
Chemical structures of five prenylated flavonoids in Yinyanghuo. The symbol “glc” refers to glucose, “rha” to rhamnose, and “xyl” to xylose.

Yinyanghuo (*Herba Epimdii*, YYH) is a popular Traditional Chinese Medicine tonic for kidney-reinforcing, used to invigorate the kidney-yang, strengthen the sinews and bones, dispel wind and eliminate dampness in clinical practice in East Asian countries for thousands of years [1,2,3]. Flavonoids as the phytoestrogens in medicinal plants have long been considered to exert beneficial effects on estrogen-related diseases by acting as selective estrogen receptor modulators. Modern pharmacological research has confirmed that flavonoids in YYH display beneficial anti-cancer, anti-depression, vasodilatation and immunoregulation properties. More than 60 flavonoids have been isolated from the herbal extracts, among which the prenylated flavonoids include icariin, epimedin A, epimedin B, epimedin C, baohuoside I (Figure 1) and so on, which are considered the main pharmacological ingredients [4,5,6].

Recently, some investigations on the metabolism of flavonoids present in YYH by the intestinal flora of rats [7,8,9], rabbits [10] and humans [11] have been carried out. It has been reported that more than 50 metabolites are discovered in plasma, bile, urine, feces after oral administration of icariin, baohuoside I, epimedin C and the herbal extracts [12,13,14,15]. However, in plasma, bile, urine, and feces, it is generally believed that intestinal flora absolutely plays a main role in the metabolism of YYH flavonoids because the intestinal microflora comprises a complex ecosystem of a large variety of bacteria which can produce negative and positive effects on metabolism, but preliminary research in our laboratory has indicated that prenylated flavonoids might be hydrolyzed both by intestinal flora and intestinal enzymes, especially lactase phlorizin hydrolase (LPH). Thus, in this experiment, we sought to explore whether the enzyme can affect the hydrolysis of prenylated flavonoids in YYH or not and investigate the effects of intestinal enzyme hydrolysis on the prenylated flavonoids in YYH.

Since intestinal enzymes and intestinal flora can hydrolyze prenylated flavonoids, and the metabolism of orally administrated prenylated flavonoids by intestinal enzymes and intestinal flora may affect their bioavailability, it is necessary to study the metabolism of prenylated flavonoids by intestinal enzymes and intestinal flora. In this study, we investigated in detail the metabolism of icariin, epimedin A, epimedin B, epimedin C, baohuoside I by rat intestinal enzyme and intestinal flora *in vitro*. We scratched the intestinal mucosa and collected the fresh feces of rats to prepare intestinal enzyme and flora incubations, using HPLC-UV to determine the metabolic rates of parent drugs and applying LC/MS/MS to figure out the chemical structures of metabolites for the purpose of exploring the metabolic pathways.

## 2. Results and Discussion

### 2.1. The Metabolic Rates of Flavonoids by Intestinal Enzyme and Intestinal Flora of Rats

**Figure 2:**
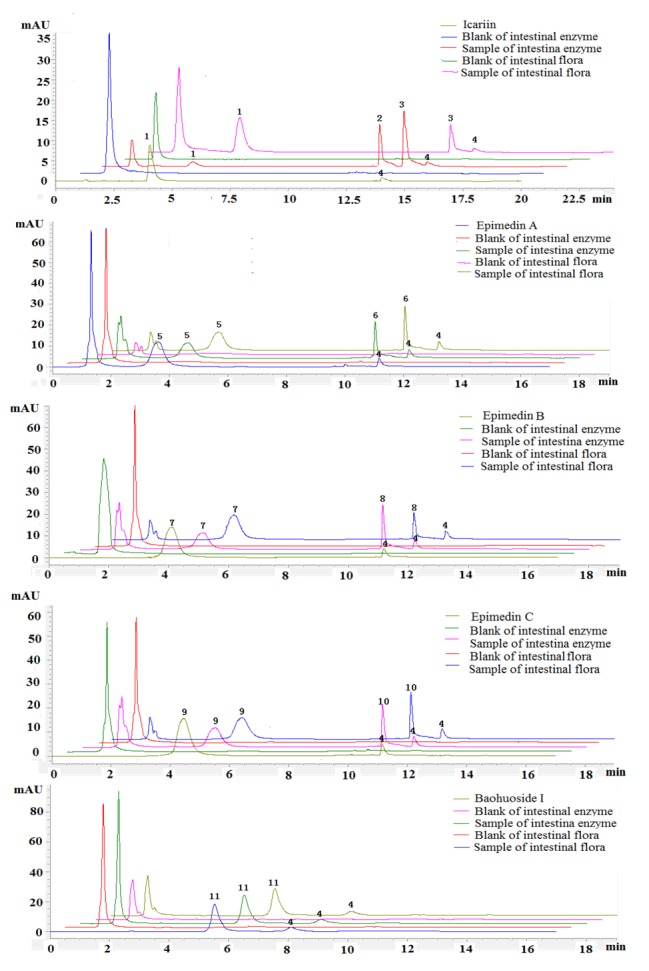
HPLC-UV elution profiles of five prenylfalvonoids and their intestinal metabolites. (1) icariin; (2) M_1_; (3) baohuoside I; (4) testosterone; (5) epimedin A; (6) M_3_; (7) epimedin B; (8) M_4_; (9) epimedin C; (10) M_5_. Testosterone was used as an internal standard (IS). M_1_, M_3_, M_4_, M_5_ were defined as the metabolites.

Five prenylated flavonoids were incubated with rat intestinal enzyme and intestinal flora solution and the degradation products were analyzed with time by HPLC-UV and LC/LC/MS. The HPLC-UV chromatograms of five prenylated flavonoids are shown in Figure 2. We can see that there were two metabolites and one metabolite created from icariin after hydrolysis by intestinal enzyme and intestinal flora, respectively, whereas epimedin A, epimedin B and epimedin C gave only one metabolite with both intestinal enzyme and intestinal flora. As to baohuoside I, no hydrolysis products were found with either intestinal enzyme or intestinal flora.

**Figure 3:**
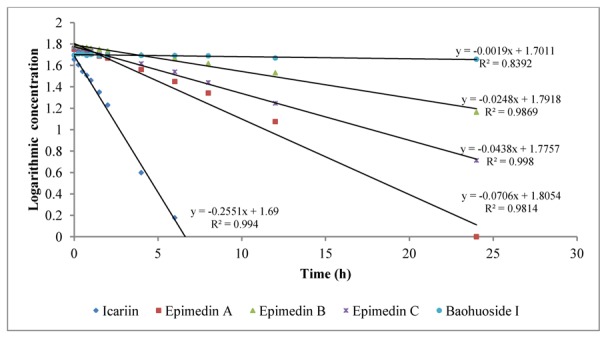
The metabolic rates of flavonoids by intestinal enzyme of rats.

**Figure 4:**
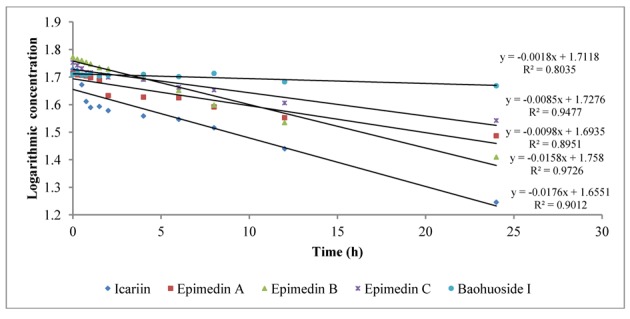
The metabolic rates of flavonoids by intestinal flora of rats.

To intuitively calculate the drug metabolic rates, the logarithmic concentrations of flavonoids (Y) and time (X/h) were used to get the corresponding regression equations. Figure 3 shows the metabolic results for intestinal enzyme and Figure 4 illustrates the metabolic results for intestinal flora. A lower slope value indicates a higher metabolic rate. Epimedin A, epimedin B, epimedin C and icariin all had higher metabolic rates with intestinal enzyme (the slope values were −0.0706 ± 0.00010, −0.0248 ± 0.00021, −0.0438 ± 0.00015, −0.2551 ± 0.00025, respectively) than with intestinal flora (the slope values were −0.0098 ± 0.00025, −0.0158 ± 0.00011, −0.0085 ± 0.00050, −0.0176 ± 0.00015, respectively). Baohuoside I had a similar metabolic rate with intestinal enzyme and flora, and the slope values were −0.0019 ± 0.00015 and −0.0018 ± 0.00011, respectively. Except for baohuoside I, the slope values for epimedin A, epimedin B, epimedin C and icariin showed significant differences between intestinal enzyme and intestinal flora (*p* < 0.05). The sequence of metabolic rates for intestinal enzyme was icariin > epimedin A > epimedin C > epimedin B > baohuoside I, and the order of metabolic rates for intestinal flora was icariin > epimedin B > epimedin A > epimedin C > baohuoside I.

Our data suggest that intestinal hydrolysis of glycosides by intestinal enzymes is rapid. Even icariin was completely metabolized in 6 h and the epimedin A was totally metabolized in 12 h in incubations with intestinal enzyme. These results inform that intestinal glycosidases are brush-border enzymes. LPH is the only mammalian brush border-glucosidase, thus, we hypothesize that LPH is responsible for the observed hydrolysis of prenylated flavonoids in YYH. LPH has two distinct catalytic active sites, one for the hydrolysis of lactose and flavonoid glucosides and another, phlorizin hydrolase, for the hydrolysis of phlorizin and –glucosylceramides [16]. As reported by Wilkinson *et al*. [17], LPH plays a major role in the deglycosylation of daidzin. In our previous research, we used gluconolactone, a LPH enzyme inhibitor, to see if LPH was involved in the hydrolysis of the flavonoids. If LPH plays an important role in absorption of icariin, epimedin A, epimdin B, epimedin C, and baohuoside I, then low activity of LPH would result in a reduced rate of metabolism.

The data of HPLC-UV show that flavonoid metabolic rates with rat intestinal enzyme were higher than those with intestinal flora. Moreover, LPH is located in the mammalian small intestine, and the intestinal flora usually exists in the large intestine. After oral administration, the drug lagged in the small intestine for a long time before it reached the large intestine, so if this situation occurs as fast in humans as we observed in the rats, the role played by intestinal flora in hydrolyzing glycosides would be significantly diminished.

### 2.2. Identification of Flavonoid Metabolites with Intestinal Flora and Intestinal Enzyme of Rats

**Figure 5:**
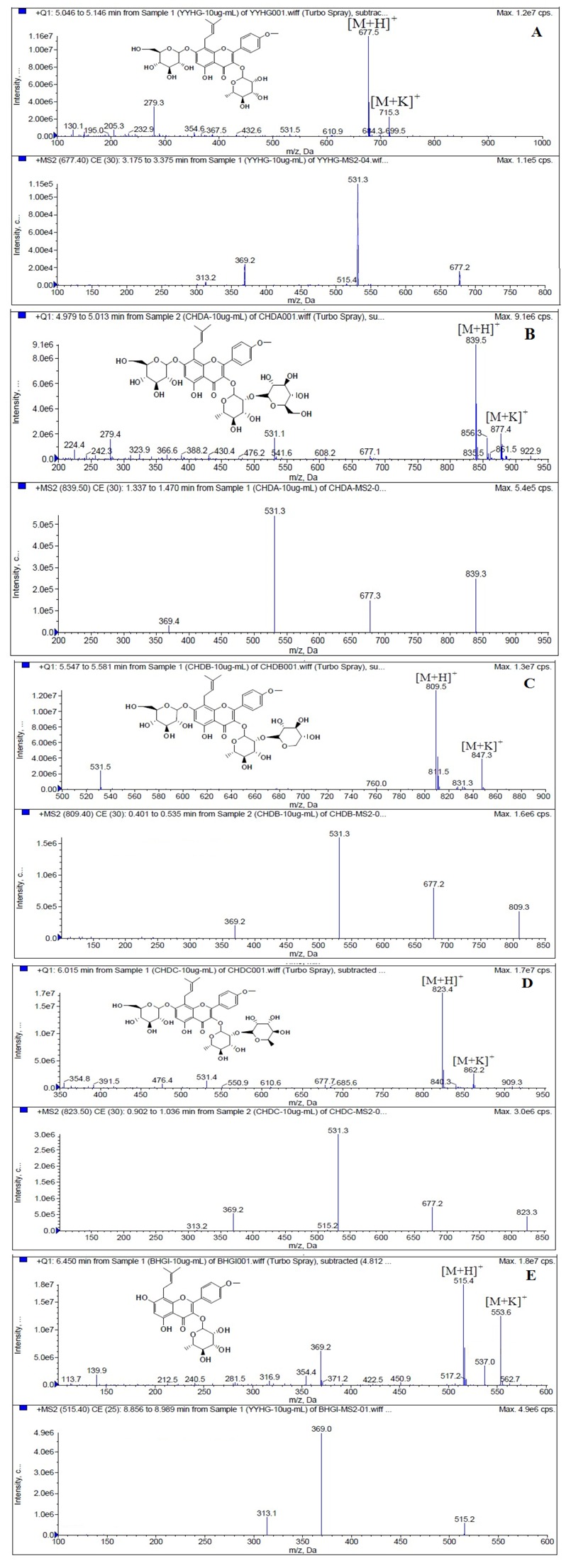
Mass spectra corresponding to prenylflavonoids. (**A**) MS and MS^2^ spectrum of icariin; (**B**) MS and MS^2^ spectrum of epimedin A; (**C**) MS and MS^2^ spectrum of epimedin B; (**D**) MS and MS^2^ spectrum of epimedin C; (**E**) MS and MS^2^ spectrum of baohuoside I.

**Figure 6:**
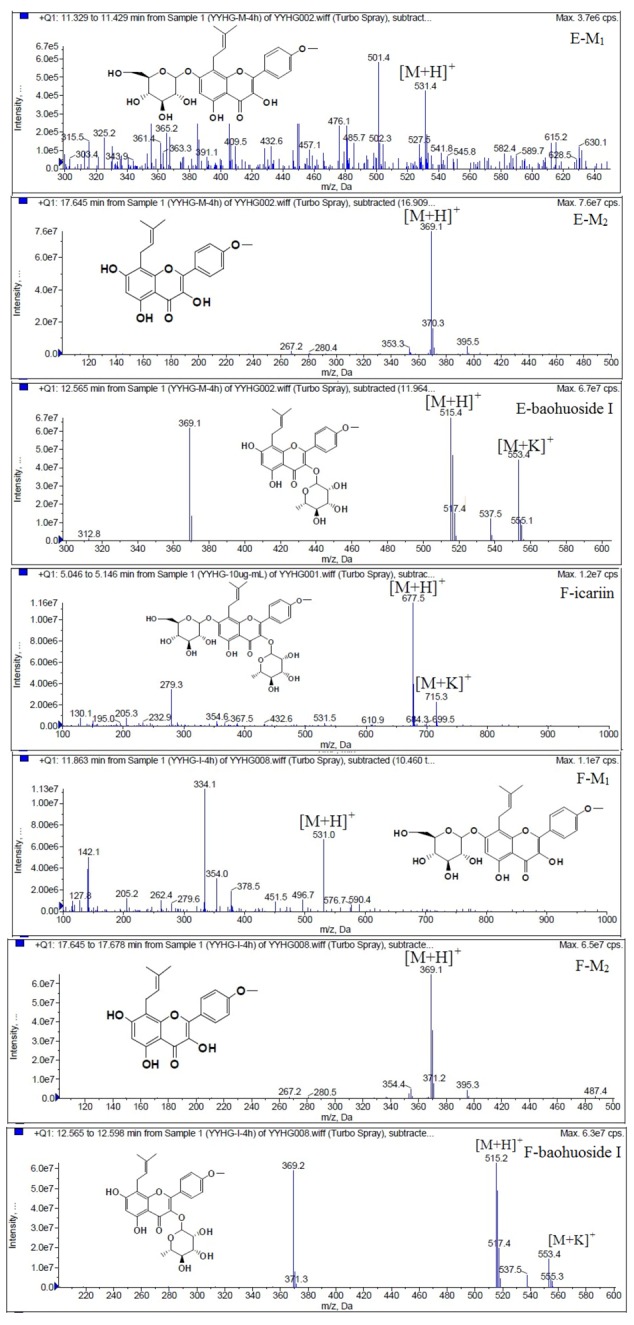
Mass spectra of metabolite of icariin. **E** for metabolites in intestinal enzyme; **F** for metabolites in intestinal flora.

**Figure 7:**
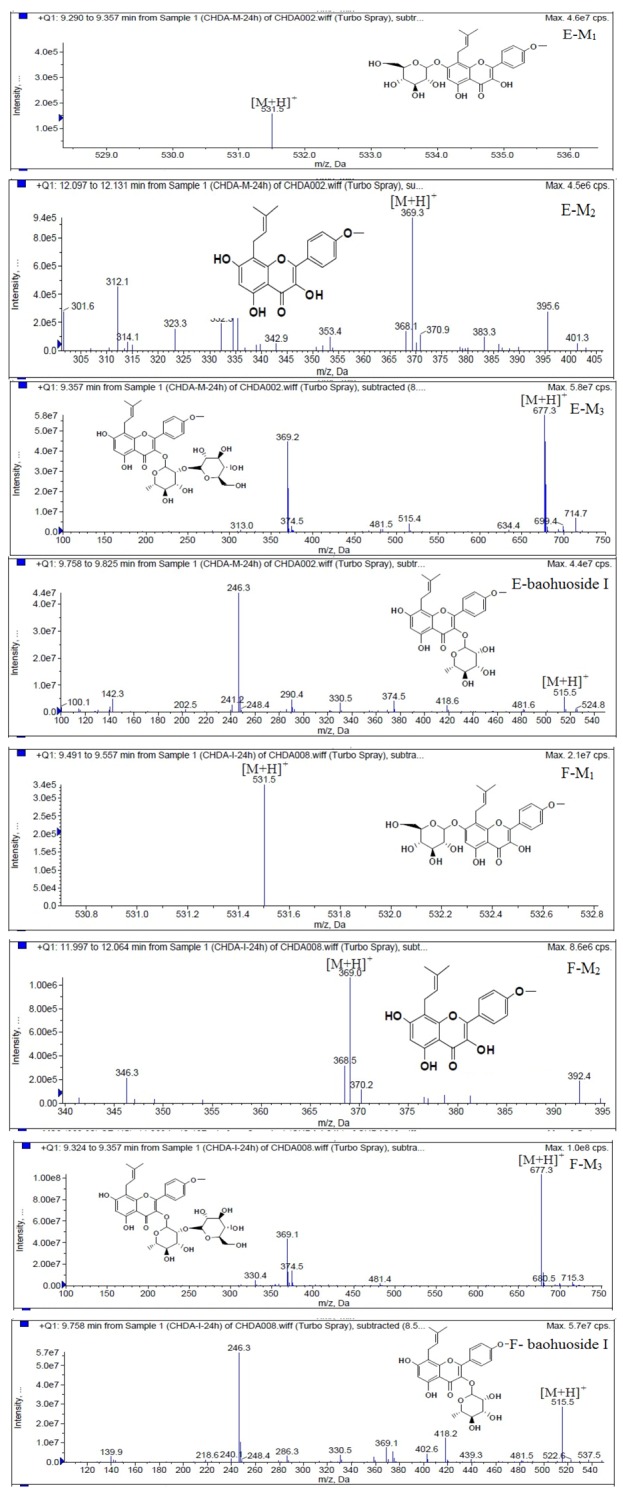
Mass spectra of metabolite of epimedin A. **E** for metabolites in intestinal enzyme; **F** for metabolites in intestinal flora.

**Figure 8:**
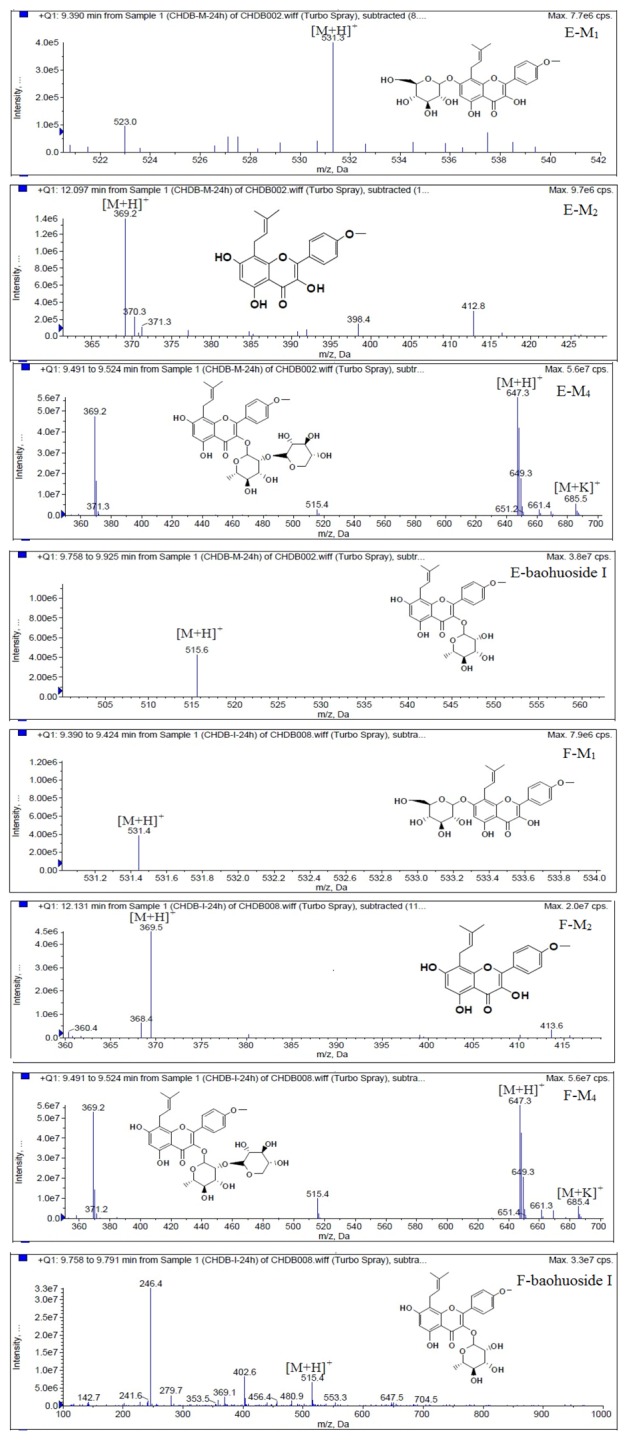
Mass spectra of metabolite of epimedin B. **E** for metabolites in intestinal enzyme; **F** for metabolites in intestinal flora.

**Figure 9:**
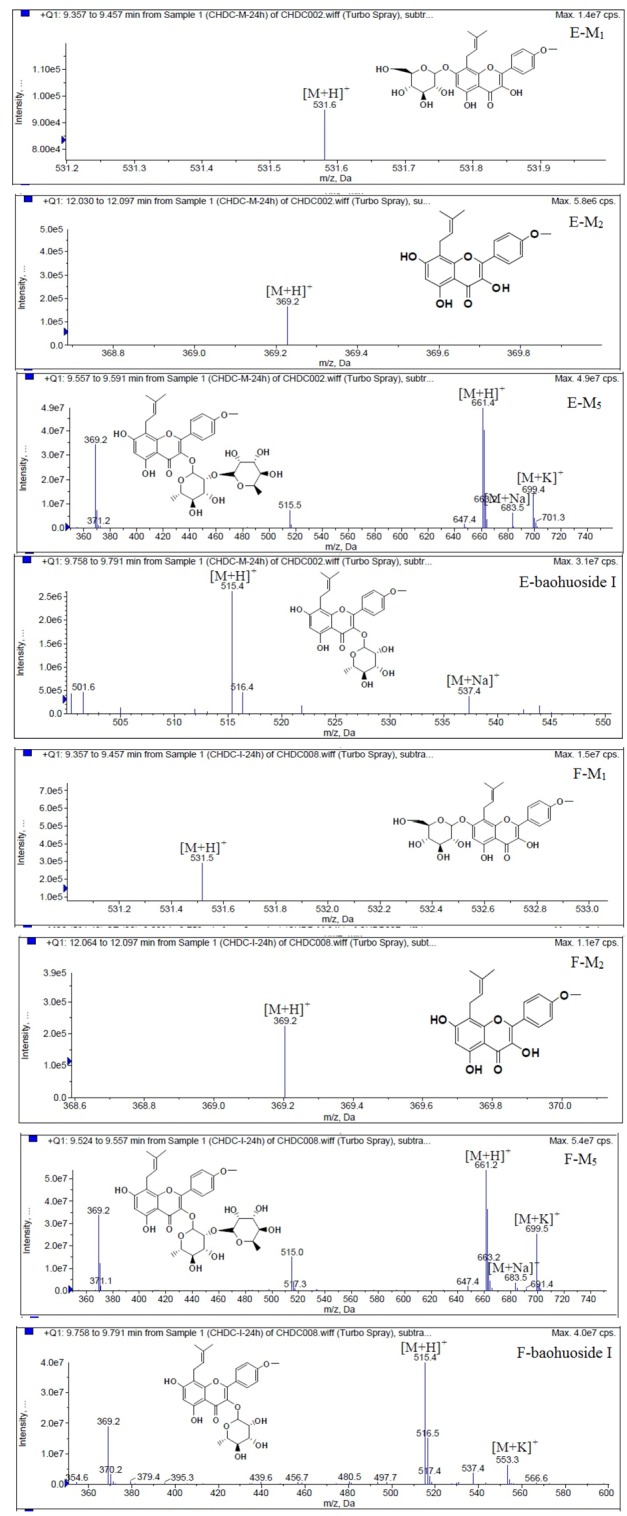
Mass spectra of metabolite of epimedin C. **E** for metabolites in intestinal enzyme; **F** for metabolites in intestinal flora.

**Figure 10:**
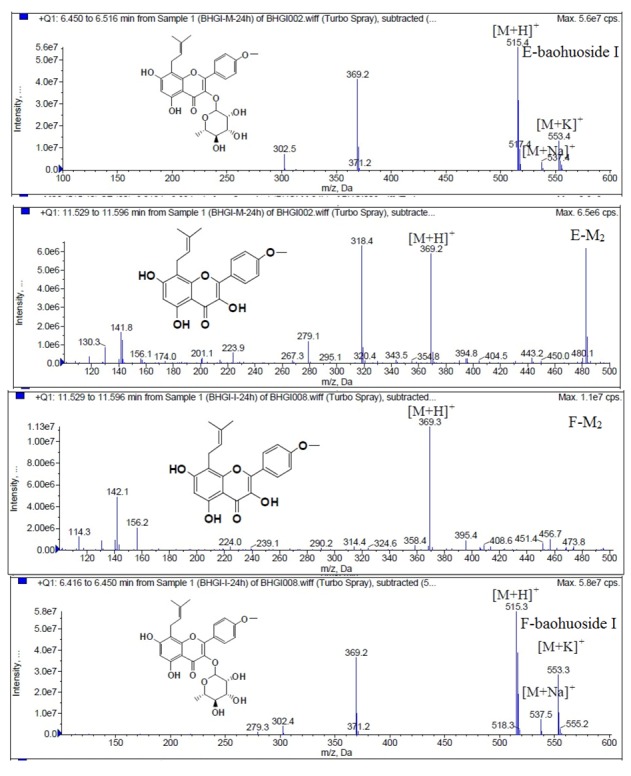
Mass spectra of metabolite of baohuoside I. **E** for metabolites in intestinal enzyme; **F** for metabolites in intestinal flora.

LC/MS/MS was used for the purpose of identifying the metabolites of the flavonoids and finding if there any other metabolites not being detected in HPLC-UV. Positive mode ESI mass spectroscopy was used in this study, [M+H]^+^ ions of sufficient abundance could be subjected to MS^n^ analysis and provided much structural information. LC-MS/MS spectra of the metabolites of icariin, epimedin A, epimedin B, epimedin C and baohuoside I were obtained via fragmentation of molecular ions were used for more precise structural identification of metabolites. The chromatographic and mass spectrometry conditions were optimized for icariin, epimedin A, epimedin B, epimedin C and baohuoside I. Full scan mass spectrum analysis of icariin, epimedin A, epimedin B, epimedin C and baohuoside I showed the presence of [M+H]^+^ at *m*/*z* 677, 839, 809, 823, 515 and [M+K]^+^ at *m*/*z* 715, 877, 847, 862, 553 respectively. The MS^2^ daughter ions spectrum of icariin, epimedin A, epimedin B, epimedin C and baohuoside I are shown in Figure 5. Fragmentation of icariin in the ion trap leaded to four product ions at *m*/*z* 313, 369, 515, 531. The most abundant product ion at *m*/*z* 531 were formed by the loss of rhamnose (146 Da). The product ion at *m*/*z* 369 was produced by the loss of rahmnose and glucose (308 Da). The product ion at *m*/*z* 515 was generated by the loss of glucose. The MS^2^ spectra of epimedin A shows three identical fragment ions at *m*/*z* 369, 531, 677. The fragment ion at *m*/*z* 369 was attributed to the loss of rahmnose and bimolecular glucose. The product ion at *m*/*z* 531 was generated through the reduction of bimolecular glucose and the ion at *m*/*z* 677 was produced via the loss of glucose. The daughter ions appearing at *m*/*z* 369, 531, 677 in the MS^2^ spectra of epimedin B were produced by the neutral loss of 440, 278, 132 Da, corresponding to the losses of glucose, rhamnose and xylose, glucose and xylose, xylose, respectively. Epimedin C yielded five main daughter ions at *m*/*z* 313, 369, 515, 531, 677. The fragment ions at *m*/*z* 369, 515, 531 and 677 were generated by the reduction of glucose and bimolecular rhamnose, glucose and rhamnose, bimolecular rhamnose, rhamnose respectively. Baohuoside I gave two product ions at *m*/*z* 313 and 369, in which the latter was attributed to the loss of rhamnose. All the product ions at *m*/*z* 313 were 56 Da less than *m*/*z* 369 by the loss of C_4_H_7_ (56 Da), which are produced by the rearrangement of the isopentene group at the position 8 of the A-ring. Above all, these characterisitic product ions and neutral losses were a sound basis to identify the metabolites of icariin, epimedin A, epimedin B, epimedin C and baohuoside I (Figure 6, Figure 7, Figure 8, Figure 9 and Figure 10).

**Figure 11:**
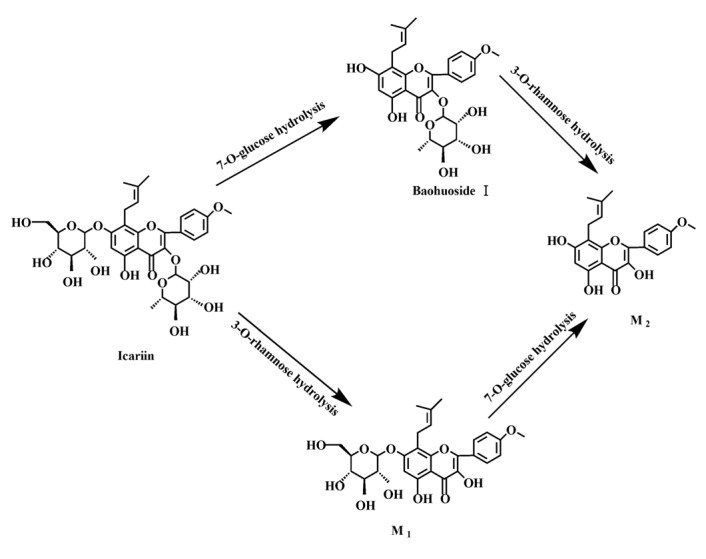
The metabolic pathway of icariin in intestinal flora and enzyme of rats.

**Figure 12:**
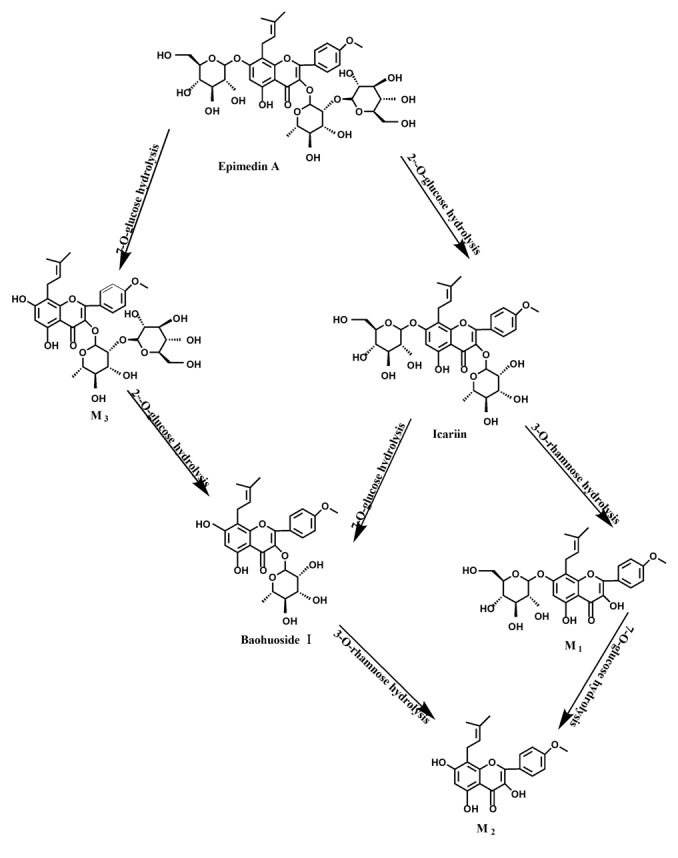
The metabolic pathway of epimedin A in intestinal flora and enzyme of rats.

**Figure 13:**
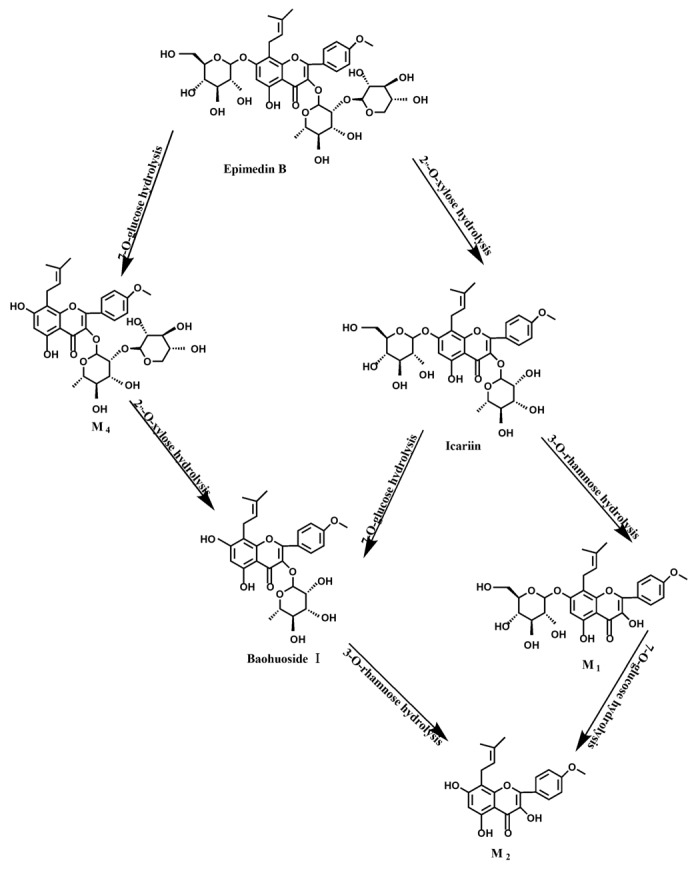
The metabolic pathway of epimedin B in intestinal flora and enzyme of rats.

**Figure 14:**
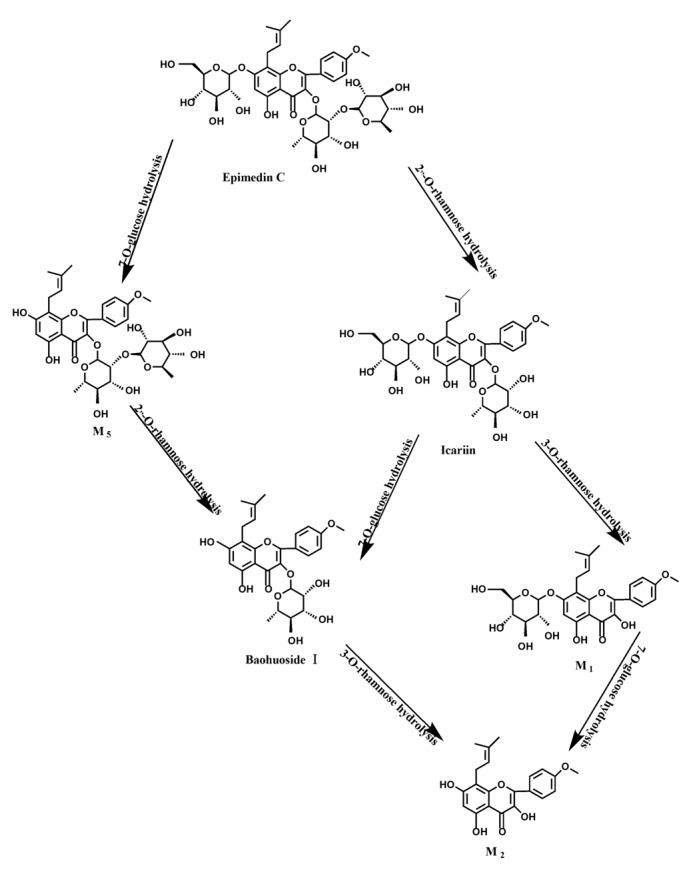
The metabolic pathway of epimedin C in intestinal flora and enzyme of rats.

**Figure 15:**
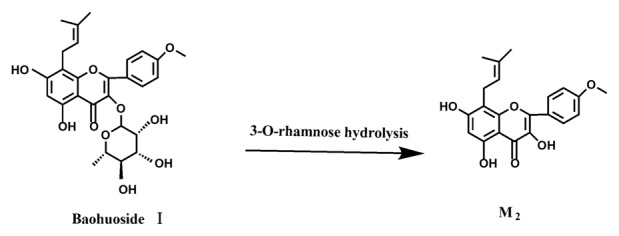
The metabolic pathway of baohuoside I in intestinal flora and enzyme of rats.

Based on our analysis, the metabolic pathways of icariin by rat intestinal flora and enzyme solution were basically the same, including 3-*O*-rhamnose, 7-*O*-glucose hydrolysis or dual 3, 7-hydrolysis (Figure 11). Compared to 3-*O*-rhamnose and dual hydrolysis of 3, 7-, the 7-*O*-glucose hydrolysis was easier. The yielded metabolites contained M_1_ (icariside I), M_2_ (icaritin), and baohuoside I. After incubation for 24 h, the parent drug, icariin, could not be found in the intestinal enzyme solution, but it was still visible in intestinal flora solution at the same time point, which demonstrated that the intestinal enzyme could more easily hydrolyze icariin. The results concluded from LC/MS/MS told that the metabolism of epimedin A by intestinal enzyme and intestinal flora were similar to each other. The metabolites included icariin, M_1_, M_2_, M_3_ (sagittatoside A) and baohuoside I, which were generated by 3-*O*-glucose-(1→2)-rhamnose hydrolysis and 7-*O*-glucose hydrolysis (Figure 12). Comparatively speaking, the 2"-*O*-glucose and 7-*O*-glucose could be removed more easily. The medicine precursor could be found in the intestinal flora of rats but not in the intestinal enzyme after incubation for 24 h. This phenomenon indicated that epimedin A was quickly metabolized by intestinal enzyme. M_1_ and M_2_ instead of icariin were detected in the incubated sample of 24 h, which demonstrated that icariin was quite easily hydrolyzed further. The sample of epimedin B incubated in intestinal enzyme and intestinal flora was determined by LC/MS/MS. Metabolic pathways by intestinal enzyme and intestinal flora were the same, including 3-*O*-xylose-(1→2)-rhamnose hydrolysis and 7-*O*-glucose hydrolysis (Figure 13). Icariin, M_1_, M_2_, M_4_ (sagittatoside B) and baohuoside I were the metabolites. The order of hydrolysis from easy to difficult is 7-*O*-glucose > 2"-*O*-xylose > 3-*O*-rhamnose. Like epimedin A, M1 and M2 replaced icariin were found in the incubated sample of epimedin B at 24 h. The conclusions summarized from LC/MS/MS data show that the metabolism of epimedin C by intestinal enzyme and intestinal flora were similar to each other. The metabolites contained icariin, M_1_, M_2_, M_5_ (2"-*O*-rhamonosylicariside) and baohuoside I, which were generated by 3-*O*-rhamnose-(1→2)-rhamnose hydrolysis and 7-*O*-glucose hydrolysis (Figure 14). Comparatively speaking, the 7-*O*-glucose could be removed more easily and 3-*O*-rhamnose was difficult to hydrolyze. M_1_ and M_2_ replacing icariin could be detected in the incubated sample at 24 h demonstrating that icariin was quite easily hydrolyzed further. This phenomenon also ocurred in epimedin A and B. Simultaneously, the content of baohuoside I in samples after incubation of epimedin C was significantly higher than that of epimedin A and B. However, the contents of M_1_ and M_2_ were so low that they could be barely be measured. Presumably these were two rhamnosyls in the molecular structure of epimedin C which made epimedin C hydrolyze more difficultly in the intestinal enzyme and intestinal flora incubations. The metabolic pathway of baohuoside I by intestinal flora was consistent with that by intestinal enzyme, which was only 3-*O*-rhamnose hydrolysis (Figure 15). After incubation for 24 h with both intestinal flora and intestinal enzyme, a high content of baohuoside I could be detected. It indicated that 3-*O*-rhamnose was hard to hydrolyze by either intestinal flora or intestinal enzyme.

Icariin is a dual glucoside, it was thought to be metabolized by hydrolysis to first produce baohuoside I after removal of 7-*O*-glucose and then baohuoside I was metabolized to produce M_2_ after further removal of 3-*O*-rhamnose. Meanwhile, icariin could be metabolized to generate M_1_ after only removal of 3-*O*-rahamnose. Epimedin A, B, C were triple glucosides with similar structures, and besides the M_3_ for epimedin A, M_4_ for epimedin B, M_5_ for epimedin C, they had other four common metabolites: icariin, M_1_, M_2_ and baohuoside I. Epimedin A was considered to be metabolized by hydrolysis to first produce M_3_ after removal of the 2"-*O*-glucose and then M_3_ was metabolized to produce baohuoside I after further removal of 3-*O*-rhamnose. Epimedin A also can be metabolized by removal of 7-*O*-glucose to then produce icariin. The generated icariin had the same metabolic pathway as above. Epimedin B was thought to be metabolized by hydrolysis to first produce icariin which had the same metabolic pathway as above after removal of 7-*O*-glucose. Epimedin B also can be metabolized by removal of 2"-*O*-oxylose to next produce M_4_ and then M_4_ was metabolized to produce baohuoside I after further removal of 3-*O*-rhamnose. Epimedin C was thought to be metabolized by hydrolysis to first produce icarrin which had the same metabolic pathway as above after removal of 7-*O*-glucose. Epimedin C also can be metabolized by removal of 3-*O*-rahmnose to secondly produce M_5_ and then M_5_ was metabolized to produce baohuoside I after further removal of 3-*O*-rhamnose. Presumably it was the presence of two rhamnosyls in the molecular structure of epimedin C which made epimedin C difficultly hydrolyzed by intestinal flora and intestinal enzyme. It can be deduced from the above data that icariin, epimedin A, B, C were generally absorbed as metabolites. On the other hand, as a single glucoside, it was difficult for baohuoside I to hydrolyze in the intestine and usually it was absorbed as the precursor.

Most of the previous studies consider that the deglycosylation of flavonoids may be caused by intestinal flora and hepatic biotransformation enzymes [18,19]. However, our study indicated that intestinal enzymes play an important role in the metabolism of the prenylated flavonoids present in YYH. Meanwhile, based on our analysis and literature data, the metabolic pathways of icariin, epimedin A, epimedin B, epimedin C and baohuoside I in intestinal flora and enzyme solution of rats were basically the same, and the absorption pathways of *Epimedium* flavonoids in rat intestine initially involved deglycosylation of flavonoid glycosides, yielding the major hydrolytic metabolites. It demonstrated that the enzyme existing in flora was a β-enzyme which had the same function as LPH in hydrolyzing *Epimedium* flavonoids. The metabolic effects in intestinal enzyme were far higher than those in intestinal flora. It might because of the expression and distribution of LPH in the upper gastrointestinal tract were comparatively large and there were no bacterial growth in the upper gastrointestinal tract.

## 3. Experimental

### 3.1. Drugs and Chemicals

Icariin (purity > 98%) was purchased from National Institute for the Control of Pharmaceutical and Biological Products (Beijing, China). Epimedin A, epimedin B, epimedin C, and baohuoside I (all purity > 98%) were provided by the Laboratory of Pharmaceutical Preparation (Jiangsu Provincial Academy of Chinese Medicine, Nanjing, China). Testosterone (purity > 98%) was purchased from Sigma-Aldrich (St. Louis, MO, USA). *Epimedium koreanum* Nakai was purchased from a drug store in Nanjing (China) and was identified to be the correct species by Professor Dekang Wu, a pharmacognosy researcher at Nanjing University of Chinese Medicine (Nanjing, China). All other materials (typically analytical grade or better) were used as received.

### 3.2. Animals

Eight weeks old Male Sprague-Dawley rats with body weight of 170–250 g were obtained from the SLEK Lab Animal Center of Shanghai (Shanghai, China), housed under standard conditions of temperature, humidity, and light. Food and water were provided ad libitum. The rats were fasted overnight before the day of the experiment. The procedures were approved by the Animal Ethics Committee of Jiangsu Provincial Academy of Chinese Medicine.

### 3.3. Preparation of Anaerobic Culture Medium

K_2_HPO_4_ (37.5 mL, 0.78%), solution A (37.5 mL, 0.47% KH_2_PO_4_, 1.18% NaCl, 1.2% (NH_4_)_2_SO_4_, 0.12% CaCl_2_, 0.25% MgSO_4_ H_2_O), Na_2_CO_3_ (50 mL, 8%), l-cysteine (0.5 g), l-ascorbic acid (2 mL, 25%), eurythrol (1 g), tryptone (1 g) and nutrient agar (1 g) were mixed together and diluted with distilled water to 1 L. Then the solution was adjusted pH to 7.5–8.0 with 2 M HCl.

### 3.4. Preparation of Intestinal Enzyme and Intestinal Flora Cultural Solution

After overnight food deprivation, rats were anesthetized by intramuscular injection of urethane (0.5 g/mL). An incision was made into the abdominal cavity to take out the small intestine and the intestine immediately was preserved in cold saline. The contents of the small intestine were removed by flushing gently with saline (0 °C) after being opened. Intestinal mucosa was blunt scratched. At the same time, fresh feces were obtained from SD rats. Intestinal mucosa and fresh feces of rats were homogenized in normal saline solution at the ratio of 1 g to 4 mL immediately, respectively. Ten mL of the filtrate of intestinal mucosa was mixed with 90 mL of cold saline to prepare 100 mL of intestinal enzyme cultural solution. Ten mL of the filtrate of fresh feces was added to 90 mL anaerobic culture medium to obtain 100 mL intestinal flora cultural solution.

### 3.5. Hydrolysis of Flavonoids in *Epimedium* by Intestinal Enzyme and Intestinal Flora

1 mL of 2 mM icariin, epimedin A, epimedin B, epimedin C and baohuoside I were added into 9 mL of intestinal enzyme and intestinal flora cultural solution, respectively. After incubation for 0, 0.25, 0.5, 0.75, 1, 1.5, 2, 4, 6, 8, 12 and 24 h at 37 °C, the incubations were extracted by a 3-fold volume of acetonitrile. Following vortexing for 1 min and centrifuging at 15,000 r/min for 15 min, 150 µL of the supernatants were evaporated to dryness under a gentle stream of nitrogen at 30 °C and reconstituted in 450 µL acetonitrile prior to injecting into the LC/MS/MS system. Similarly, 400 µL aliquots of the supernatants were mixed with 100 µL of 100 µM internal standard testosterone which dissolved in acetonitrile for HPLC-UV analysis [20,21].

### 3.6. HPLC-UV Analysis of Samples Incubated in Intestinal Enzyme and Intestinal Flora

The conditions for HPLC-UV analysis of flavonoids in hydrolysis samples were as follows: system, Agilent 1260 HPLC-UV with photodiode array detector; column, ZORBAX SB-C18, 5 µm, 4.6 × 250 mm (Agilent, Santa Clara, CA, USA); mobile phase A, water; mobile phase B, acetonitrile; gradient, 0–6.5 min: 70% A, 6.5–10 min: 45% A, 10–14 min: 45% A, 14–15 min: 70% A; flow rate, 1 mL/min; wavelength 270 nm for icariin, epimedin A, epimedin B, epimedin C and baohuoside I and 254 nm for internal standard testosterone; injection volume, 10 µL. The HPLC-UV chromatograms of samples are shown in Figure 2. In general, these methods were selective and reproducible with day to day variability less than 3%. The accuracy and precision were greater than 98%. The tested linear response ranges for all flavonoids were 6.25 to 100 µM, respectively. The retention times for icariin, M_1_, baohuoside I, epimedin A, M_3_, epimedin B, M_4_, epimedin C, M_5_ were 4.1, 12.3, 14.5, 3.9, 10.1, 4.1, 10.2, 4.2, and 10.1 min, respectively. And the retention times for testosterone in different solutions were 13.5 (in icariin), 11.3 (in epimedin A), 11.3 (in epimedin B), 1 1.3 (in epimedin C).

### 3.7. LC/MS/MS Identification of Metabolites

Different hydrolysis samples were analyzed by LC/MS/MS using a system of API 4000 triple quadrupole mass spectrometer (AB SCIEX, Carlsbad, CA, USA) equipped with an electrospray ionization (ESI) source. The samples were separated on a Zorbax SB-C_18_ column (250 mm × 4.6 mm, 5 µm, Agilent). The mobile phase consisted of water containing 4% acetic acid (A) and acetonitrile (B). The gradient program of icariin was as follows: 0 to 8 min, 70% A, 8 to 10 min, 70% to 45% A, 10 to 15 min, 45% A, 15 to 16 min, 45% to 0% A, 16 to 20 min, 0% A, 20 to 21 min, 0% to 70% A; the gradient program of baohuoside I was as follows: 0 to 7 min, 55% A, 7 to 8 min, 55% to 25% A, 8 to 15 min, 25% A, 15 to 16 min, 25% to 0% A, 16 to 20 min, 0% A, 20 to 21 min, 0% to 55% A; the gradient programs of epimedin A, epimedin B and epimedin C were as follows: 0 to 7 min, 72% A, 7 to 8 min, 72% to 25% A, 8 to 15 min, 25% A, 15 to 16 min, 25% to 0% A, 16 to 20 min, 0% A, 20 to 21 min, 0% to 72% A, and the flow-rate was 0.7 mL/min.

For MS analysis, the ESI source was operated in the positive ion mode. The MS operating parameters were as follows: ionization voltage 4.5 kV; temperature 500 °C; nebulizer (nitrogen) pressure 35 psi; turbo gas (nitrogen) pressure 35 psi; curtain gas (nitrogen) pressure 10 psi; collision gas (nitrogen) pressure 6 psi; multireaction monitoring mode (MRM). The LLOQ for icariin, epimedin A, epimedin B, epimedin C, baohuoside I were 1.05, 1.89, 1.56, 1.86, 1.16 µM, respectively.

### 3.8. Data Analysis

One-Way ANOVA with Tamhane’s post hoc analysis was used to analyze data for multiple comparisons when the variance was shown to be unequal, Unpaired Student’s *t* test (Microsoft Excel) was used to analyze the data when there were only two groups in the experiments. The prior level significance was set at *p* < 0.05.

## 4. Conclusions

This paper compared the metabolism of *Epimedium* flavonoids by intestinal enzyme and intestinal flora extracts. It first proposed the idea that the intestinal enzyme and intestinal flora jointly controlled the metabolism of *Epimedium* flavonoids, and more importantly, that intestinal enzyme played a more important role than intestinal flora. The metabolism controlled by intestinal enzyme is the key step in the metabolic processes of icariin, epimedin A, epimedin B, epimedin C, and baohuoside I. In future research the effects of intestinal enzymes, especially LPH, should be emphasized.
